# AGE MODERATES THE ASSOCIATION BETWEEN ENGAGEMENT IN MINDFULNESS AND PSYCHOLOGICAL DISTRESS AMONG CANCER SURVIVORS

**DOI:** 10.1093/geroni/igz038.2387

**Published:** 2019-11-08

**Authors:** Anao Zhang, Rita X Hu, Lydia Li, Kaipeng Wang

**Affiliations:** 1 University of Michigan, Ann Arbor, Michigan, United States; 2 University of Michigan, Ann Arbor, Ann Arbor, Michigan, United States; 3 Texas State University, San Marcos, Texas, United States

## Abstract

Geriatric cancer survivors constitute up to 65% of the U.S. survivorship population, and their psychosocial wellbeing are increasingly recognized. Facing high risk of psychological distress, many cancer survivors engage in mindfulness to reduce psychological distress. However, the acceptance of mindfulness across age groups was rarely examined. Based on the Motivational Theory of Life Span Development, mindfulness practice actives survivors’ secondary control strategies to better cope with the loss of primary control caused by cancer. In other words, older survivors may respond differently than their young counterparts when facing psychological distress. Therefore, it is important to examine to what extent engagement in mindfulness is different across age groups. Using the cross-sectional 2017 National Health Interview Survey (N=3,068), this study evaluated the association between engagement in mindfulness and psychological distress among cancer survivors and examined whether age moderates such relationship. Age significantly moderated the association between psychological distress and engagement in mindfulness, OR=0.97, p<0.05. The correlation coefficient between engagement in mindfulness and psychological distress were significantly greater among younger cancer survivors (<65 years old) than their older counter parts (65+), z=2.24, p<0.05. Consistent with previous literature and the Motivational Theory, this study found age moderates the relationship between psychological distress and mindfulness engagement, and this relationship was much stronger among younger cancer survivors comparing to their older counter parts. Therefore, geriatric cancer survivors’ unique psychosocial challenges should be addressed in ways that are appropriate to their developmental characteristics.

